# Ozone as Anæsthetic and Hypnotic

**Published:** 1883-09

**Authors:** 


### OZONE AS ANÆSTHETIC AND HYPNOTIC.

Prof. Binz, of Bonn, has made a series of experiments upon the physiological effects of pure ozonized air. He did not prepare the ozone which he employed by chemical means, as ozone prepared in this way contains many impurities, but by electricity, using a tube made by Werner Siemens for the silent discharge. The tube was an inch in diameter and a foot long, and was operated with four Bunsen cells, and an induction cell that would give a spark nearly an inch long when the battery was in good order.

The ozone tube was connected with a chloride of calcium cylinder, charged with eight inches of coarsely powdered chloride of calcium between plugs of glass wool. The air to be ozonized had to pass through this tube, which filtered and dried it sufficiently; the former is of importance for the purity of the ozone, the latter for the quantity.

The ozone thus prepared, when conducted into water recently distilled over permanganate of potash and then made slightly alkaline, did not show a trace of nitrous or nitric acid. A second experiment gave the same result.

We cannot go into all the details of the precautions used in its inhalation and the apparatus employed. Experiments made upon the lower animals showed that an apparent sleeping state could be produced before the air passages were irritated by it, and this was more distinctly noticed in men. The breathing before sleep began was quiet and full; the persons experimented upon said that it was easy and comfortable, and the passage from the waking to the sleeping state was a feeling of the most agreeable indifference. The pulse never exhibited any perceptible change during the experiment, nor was there any alteration in the pupil of the eye or the color of the face. If the quantity of ozone inhaled is too large, from the apparatus working too fast or the tube being too near the nostrils, it may excite very violent coughing, nausea and choking, but not the slightest sensation of local irritation in the chest is perceived.

In all observations hitherto made as to the effect of ozone on men, they have only described the irritating effect on the air passages, resembling those of chlorine. The reason of this was that the ozone was not mixed with air in suitable proportions, and in most cases also to impurities in the ozone used. In the former respect Binz compares ozone to alcohol, which used in its concentrated form irritates the mucous membranes violently, destroys the epithelium, coagulates albumen, etc., but when very dilute scarcely exerts any perceptible influence on them.

Owing to the very transitory effect of ozone, it will never take the place of nitrous oxide for anæsthesia for surgical purposes. Binz himself does not lay much weight upon the practical importance of the ozone sleep, but hints that further experiments in this direction may lead to important results.—*Pharm. Centralhalle.*

Physicians are aware that the air of mountains exercises a hypnotic and exhilirating effect, probably due to the larger proportion of ozone which it contains. Has any one experimented with the latter as a remedy for insomnia ?—EDITOR.

